# Use of intravenous antibiotics for the management of suspected chorioamnionitis: insights from women's wellness and research center

**DOI:** 10.1080/20523211.2026.2664880

**Published:** 2026-05-07

**Authors:** Somaya Koraysh, Alaa Ismail, Ruba Mahmoud Shaker, Mohamed Abdelaziz, Muna Al-Saadi

**Affiliations:** Department of Pharmacy, Hamad Bin Khalifa Medical City, Doha, Qatar

**Keywords:** Antibiotics, antimicrobial stewardship, chorioamnionitis, obstetrics, pharmacy interventions, pregnancy

## Abstract

**Background**: Chorioamnionitis significantly impacts both maternal and neonatal outcomes. Due to the critical need for rapid management, coordinated interprofessional collaboration is essential to ensure prompt administration of intravenous (IV) antibiotic therapy. Hospital pharmacists hold a strategic capacity to enhance clinical outcomes and reinforce safe medication practices.

**Aim**: To describe IV antibiotics prescribing patterns and assess their clinical appropriateness in the treatment of suspected chorioamnionitis at Women Wellness and Research Center (WWRC), evaluate antibiotic-focused pharmacy interventions, and propose evidence-driven strategies to address identified challenges.

**Method:**: A retrospective chart review of IV antibiotics used for females admitted for labor between 01/07/2024 and 31/12/2024 was conducted. Pharmacists’ intervention notes related to prescribed antibiotics were collected and analyzed. Descriptive reporting of findings will be provided.

**Results**: A total of 464 pregnant patients were included. Majority were Arab-speaking (55.2%) and 63.1% were primigravida (n = 293). Modes of delivery included cesarean section (34.9%), normal delivery (31.5%), and assisted normal delivery (22.8% vacuum-assisted, 9.7% forceps-assisted). Only 376 (81.4%) of the reported 460 cases met the diagnostic criteria for suspected chorioamnionitis. All patients included received gentamicin, 97.8% received ampicillin (mean duration 2.8 ± 0.7 days), 15.5% received clindamycin (0.5 ± 0.8 days), 20.5% received metronidazole (2.5 ± 1.7 days), and 3.4% received ceftriaxone (2.4 ± 1.2 days). Usage of more than 2 antibiotics was reported in 135 patients (29%). The most reported intervention performed by IV staff and clinical pharmacists was “dosing/ administration” (68.2% and 31.1%, retrospectively). Gentamicin dosing was corrected in 306 prescriptions (65.9%) out of the 464. Gentamicin dosing algorithm and suspected chorioamnionitis diagnosis checklist have been proposed in this work.

**Conclusion**: Observed deviations from recommended guideline-concordant antibiotic use reveal opportunities for system-level optimization initiatives. The findings underscore pharmacists’ crucial contributions in intercepting medication-related inaccuracies and advancing sustainable error-reduction strategies to enhance obstetric antimicrobial stewardship and improve patient safety.

## 1. Background

Chorioamnionitis is an intra-amniotic infection typically resulting from an ascending polymicrobial infection (Conde-Agudelo et al., 2020; Tita, n.d). It has been reported that the incidence of chorioamnionitis is 3.9% of births, with a higher incidence rate in preterm births (Tita). Clinical manifestations of chorioamnionitis include maternal fever (>38°C), uterine tenderness, maternal (>100 bpm) and/or fetal (>160 bpm) tachycardia, leukocytosis, and occasionally malodorous amniotic fluid. Chorioamnionitis is primarily diagnosed clinically, with the support of laboratory and microbiological findings where permitted (Committee Opinion No. 712, 2017; Tita, n.d).

Acquiring chorioamnionitis can have a significant impact on maternal outcomes, including endometritis, wound infections, pelvic abscess or sepsis, increased need for caesarean section, postpartum haemorrhage, and increased length of hospital stay (Committee Opinion No. 712, 2017; Lukanović et al., 2023). Neonatal consequences have also been reported, including preterm birth, early-onset sepsis, respiratory complications, and neonatal mortality (Conde-Agudelo et al., 2020; Lukanović et al., 2023). These risks underscore the need for rapid recognition and appropriate therapeutic intervention.

Prompt identification, facilitating delivery, and empirical intrapartum intravenous (IV) antibiotic therapy are the cornerstones of chorioamnionitis management in order to prevent the aforementioned complications (Committee Opinion No. 712, 2017). Broad-spectrum antibiotics are utilised empirically to cover the polymicrobial pathogens commonly involved in the cervicovaginal migration process. First-line therapy consists of ampicillin and gentamicin, with the addition of clindamycin or metronidazole if caesarean delivery is performed (Committee Opinion No. 712, 2017). Intravenous (IV) antibiotics can be continued for up to 24 h after caesarean delivery; however, for vaginal births, treatment is typically restricted to the intrapartum period, given the absence of evidence to support any added benefit of post partum therapy (Committee Opinion No. 712, 2017; Lukanović et al., 2023; Tita, n.d).

Given the urgent nature of prompt diagnosis and treatment, the healthcare team is expected to communicate and work seamlessly to ensure the timely administration of antibiotics to the patient. It is established that the well-coordinated collaboration efforts among doctors, nurses, and pharmacists can optimise therapeutic plans, minimise medical errors, and guarantee holistic patient care (Lee et al., 2024; Satsuma et al., 2020). Hospital pharmacists, with their deep involvement in the medication use processes, are uniquely positioned to optimise patient care and ensure patient safety (Naseralallah et al., 2024; Naseralallah et al., 2025; Naseralallah et al., 2024).

In the Women Wellness and Research Centre (WWRC), IV antibiotics are prepared by a team of pharmacists working in an IV and admixture preparation suite. The team is trained and qualified to validate and prepare all IV admixtures and send them to the assigned patients in a timely manner. They are responsible for identifying and rectifying any errors related to IV prescriptions, thus ensuring the delivery of the right medicine with the right dose, frequency, and instructions to the right patient, contributing to optimum health care.

The purpose of this study is to describe the prescribing behaviour and appropriateness of IV antibiotics administered to patients admitted to WWRC who were diagnosed with chorioamnionitis intrapartum. The study will also shed light on the potential role of pharmacists in managing IV antibiotics utilised for the treatment of chorioamnionitis and will address potential issues related to the appropriateness of medications.

## 2. Methods

### 2.1. Ethics approval

Ethics approval for the study was obtained from the Medical Research Centre (MRC) at Hamad Medical Corporation (HMC) in 2024 (MRC-01-25-996).

### 2.2. Patient pathways and management

Pregnant patients admitted to WWRC for labour who develop symptoms suggestive of chorioamnionitis are primarily treated with ampicillin 2 g every 6 h and gentamicin 1.5 mg/kg every 8 h (using adjusted body weight when total body weight is more than 1.2 times the ideal body weight) (ACOG, 2024; Drew, 2024). In case of penicillin allergy, clindamycin is used as an alternative to ampicillin. For patients undergoing caesarean section (c-section), clindamycin (a single dose of 900 mg preoperatively or 900 mg every 8 h) and/or metronidazole (500 mg every 8 h) are used. The typical treatment pathway includes the confirmation of the chorioamnionitis diagnosis by the physician, followed by the assigned nurse activating the treatment protocol by contacting the IV pharmacy suite for prompt verification of orders through the electronic system and supply of IV antibiotics. All dosage adjustments are typically initiated by IV staff pharmacists contacting prescribing physicians to modify the orders, regardless of whether the pharmacist recorded the intervention on the electronic health record system (CERNER) or not. All IV medications’ surveillance and management are primarily conducted by the IV staff pharmacy team, with the clinical pharmacy team providing support when needed.

Staff pharmacists differ from clinical pharmacists in their roles and experience. While staff pharmacists manage mainly operational roles alongside their basic clinical roles, clinical pharmacists are fully immersed within the multi-disciplinary team providing direct clinical care to admitted patients. In an effort to expand their roles, IV staff pharmacists in our institution have received specialised training and are well-equipped to clinically and operationally handle IV admixture.

### 2.3. Study design and setting

A retrospective chart review of IV antibiotics used for females admitted for labour in WWRC between 01 July 2024 and 31 December 2024 was conducted. The study identified the usage of IV antibiotics in patients with suspected chorioamnionitis during the intrapartum period. Pharmacists’ intervention notes (embedded in the Cerner system) on the mentioned antibiotics were collected and analysed.

All patients admitted for delivery in WWRC during the study period who received either ampicillin or gentamicin were included in the study with no restrictions. Patients receiving ampicillin or gentamicin preoperatively as prophylaxis were excluded. Data regarding the use of penicillin G during hospital stay was not collected. Follow-up data on the administration of oral antibiotics on discharge were not collected. All pharmacist interventions documented at the time of receiving the antibiotics were included.

Given the explorative nature of this study on IV antibiotics prescribing behaviour in the said population, as well as the absence of a comparative objective, all patients meeting the eligibility criteria were included.

For the assessment of appropriateness of the chorioamnionitis diagnosis, we followed the American College of Obstetrics and Gynaecology (ACOG) definition, stating that the diagnosis of suspected chorioamnionitis is made when the maternal temperature is ≥39.0°C or when the maternal temperature is 38.0–38.9°C and one additional clinical risk factor (uterine tenderness, maternal (>100bpm) and/or fetal (>160bpm) tachycardia, leukocytosis, malodorous amniotic fluid) is present (ACOG, 2024).

### 2.4. Data processing

Patient demographics (age, gender, parity, gravidity, nationality, weight, and height), reported signs and symptoms of chorioamnionitis (maternal fever >38°C, uterine tenderness, maternal tachycardia >100 bpm and/or fetal tachycardia >160 bpm, leukocytosis, and malodorous amniotic fluid), medication regimens used for treatment (drug, dose, frequency, and duration), and clinical pharmacy interventions were collected for eligible patients.

Further manual review of physicians and labor and delivery nurse notes was conducted if required information was not easily retrieved. The collected data was anonymized and processed using Microsoft Excel. Data cleansing was performed to remove duplicate entries, incomplete records, or unclear documentation.

### 2.5. Data analysis

Data collected was analysed using Statistical Package for Social Sciences (SPSS®) version 21 (IBM Statistics for Windows; IBM Corp, Armonk, New York, USA). A descriptive approach was utilised to report on the intervention numbers, types, and severity. Frequencies and percentages were used to summarise the responses generated from categorical variables, while continuous data were presented as mean ± standard deviation (SD) or median (IQR).

## 3. Results

### 3.1. Baseline characteristics

Table 1 displays the baseline characteristics of the patients included. The study comprised 464 pregnant patients, with a mean age of 30.5 ± 4.5 years, an average weight of 83.5 ± 9.7 kg, and an average height of 158.9 ± 4.4 cm. Arabic-speaking residents constituted the majority of the cohorts (70.5%). Of the included pregnancies, 63.1% were primigravida (n = 293), 31.1% had 2–4 pregnancies, and only 27 patients (5.8%) were grand multigravida. Of the current cohort, 18.3% (n = 85) had a history of abortion. The mode of delivery performed ranged from caesarean section (34.9%), normal delivery (31.5%), to assisted normal delivery (22.8% vacuum-assisted, 9.7% forceps-assisted).

**Table 1.** Baseline characteristics of included patients (n = 464).

Age (mean, SD)	30.5	4.5
Weight (mean, SD)	83.5	9.7
Height (mean, SD)	158.9	4.4
History of abortion	85	18.3%
Gravida		
1	293	63.1%
2–4	144	31.1%
≥ 5	27	5.8%
Nationality		
Arabic-speaking residents and nationals	327	70.5%
Non-Arabic-speaking residents	137	29.5%
Mode of delivery		
Caesarean section	162	34.9%
Normal delivery	146	31.5%
Vacuum-assisted vaginal delivery	106	22.8%
Forceps-assisted vaginal delivery	45	9.7%
Miscarriage or termination of pregnancy	4	0.9%
NA	1	0.2%

### 3.2. Clinical manifestations and diagnostic criteria

Table 2 reports on the clinical diagnosis of included patients. Indications reported in patients’ profiles included chorioamnionitis (n = 460, 99.2%). Other diagnoses included post-partum fever (0.2%), urinary tract infection (0.2%), incomplete septic miscarriage (0.2%), and positive wound culture (0.2%).

**Table 2.** Clinical diagnosis and behaviour of antibiotics use.

**Indication for antibiotics**		
Chorioamnionitis	460	99.2%
Post-partum fever	1	0.2%
Urinary tract infection	1	0.2%
Incomplete septic miscarriage	1	0.2%
Wound culture	1	0.2%
**Chorioamnionitis charted on system**	58	12.6%
**Clinical chorioamnionitis diagnosis met (n** **=** **460)**	376	81.7%
Maternal fever (>37.8)	383	83.1%
Maternal Tachycardia (>100 bpm)	364	79.1%
Leukocytosis (WBC>15)	350	76.1%
Fetal tachycardia (>160 bpm)	292	63.5%
Foul-smelling amniotic fluid (or meconium-stained)	104	22.6%
Uterine tenderness	2	0.4%
**Antibiotics used**		
Ampicillin	454	97.8%
Gentamicin	464	100.0%
Clindamycin	72	15.5%
Metronidazole	95	20.5%
Ceftriaxone	16	3.4%
**Duration of antibiotics (mean days, SD)**		
Ampicillin	2.8	0.7
Gentamicin	2.8	0.7
Clindamycin	0.5	0.8
Metronidazole	2.5	1.7
Ceftriaxone	2.4	1.2
**Change in gentamicin dose**	306	65.9%
**Documented pharmacist intervention**s **for dose changes**	175	57.2%

Although 460 cases were reported as suspected chorioamnionitis as the primary diagnosis, only 376 (81.7%) met the criteria for diagnosis (Table 2). The most reported manifestations were maternal fever, maternal tachycardia, and leukocytosis (83.1%, 79%, and 76.1%, respectively). Fetal tachycardia was reported in 63.5% of cases, and meconium/ foul smelling ammonitic fluid was present in 22.6% of cases. Only 2 charts reported uterine tenderness as part of the diagnostic criteria. Despite having 376 patients meeting the diagnostic criteria, physicians had official documentation of the diagnosis in only 58 patients’ charts.

### 3.3. Prescribing pattern of IV antibiotics peripartum

IV antibiotic usage is described in Table 2. All included patients received gentamicin (n = 464), and 97.8% received ampicillin (n = 454). Of the included patients, 135 have received more than two antibiotic types during their admission (29%). Clindamycin (n = 72, 15.5%) was utilised in cases of penicillin allergies (n = 8) or occasionally as an add-on prophylaxis dose prior to c-section (n = 64). Metronidazole was added to ampicillin/gentamicin in 95 cases (20.5%), 65 of which were post c-section. It was noted that 26 patients received a single clindamycin dose preoperatively, followed by a course of metronidazole post operatively.

Ceftriaxone was received by 16 patients (3.4%); indications include suspected urinary tract infection (n = 2), post-partum fever (n = 2), post operative infection (n = 2), postpartum haemorrhage (n = 3), not a chorioamnionitis case (n = 2), suspicion of infection before diagnosing chorioamnionitis (n = 3), allergy to gentamicin (n = 1), and undocumented reason (n = 1).

Ampicillin and gentamicin were continued for a mean duration of 2.8 days (± 0.7), with only 10 patients completing a treatment course of 4 to 7 days. Metronidazole was given for an average of 2.5 ± 1.7 days, ceftriaxone for 2.4 ± 1.2 days, while clindamycin was usually given for 1 to 2 doses only (mean days 0.5 ± 0.8) (Table 2).

### 3.4. Pharmacists' role and interventions on prescribing behaviour

**Figure 1.**
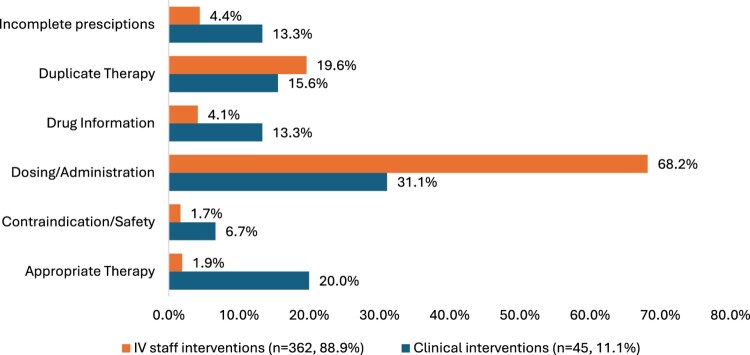
Types of interventions performed by IV staff team vs. clinical pharmacy team.

Table 3 and Figure 1 show the characteristics and types of interventions performed by IV staff pharmacists and clinical pharmacists. As expected, the majority of interventions were documented by IV staff pharmacists (n = 362, 88.9%), while clinical pharmacists accounted for 45 interventions (11.1%).

**Table 3.** Characteristics of interventions made by pharmacists (n = 407).

Clinical Pharmacists	45	11.1%	IV staff pharmacists	362	88.9%
Appropriate Therapy	9	20.0%	Appropriate Therapy	5	1.4%
*Additional Therapy Required*	5	55.6%			
*Alternative Therapy*	3	33.3%	*Medication without Indication*	1	20.0%
*Appropriate Laboratory recommended*	1	11.1%	*Therapeutic Drug Therapy*	4	80.0%
Contraindication/Safety	3	6.7%	Contraindication/Safety	7	1.9%
*Allergy*	2	66.7%	*Allergy*	7	
*Breastfeeding*	1	33.3%			
Dosing/Administration	14	31.1%	Dosing/Administration	248	68.2%
*IV to PO*	5	35.7%	*Dose adjustment/ Calculation*	188	75.8%
*Optimise frequency*	4	28.5%	*Optimise frequency*	57	22.9%
*Optimum Administration*	2	14.3%	*Optimum Administration*	3	1.3%
*Dose Calculation*	2	14.3%			
*Hold/Resume*	1	7.1%			
Drug Information inquiry	6	13.3%	Drug Information inquiry	15	4.1%
Duplicate Therapy	7	15.6%	Duplicate Therapy	71	19.6%
Incomplete prescriptions	6	13.3%	Incomplete prescriptions	16	4.4%
*Missing duration*	1	16.7%	*Missing dose*	1	6.3%
*Missing indication*	5	83.3%	*Missing duration*	4	25.0%
			*Missing frequency*	1	6.3%
			*Missing indication*	8	50.0%
			*Missing route*	1	6.3%
			*Missing weight*	1	6.3%

The most common type of intervention performed by IV staff and clinical pharmacists was ‘dosing/administration' (68.2% and 31.1%, respectively). Specifically, IV staff pharmacists intervened to aid with dose calculation and adjustment (n = 188, 76.1%), followed by frequency adjustment (n = 56, 22.7%), and administration (n = 3, 1.2%). In contrast, clinical pharmacists recommended IV to PO change (n = 5, 35.7%), followed by frequency adjustment (n = 4, 28.5%), administration-related interventions (n = 2, 14.3%), dose calculation (n = 2, 14.3%), and requests to hold or resume therapy (n = 1, 7.1%). Notably, the dose of gentamicin was corrected in 306 prescriptions out of the 464 (65.9%), yet only 175 (57.2%) of these corrections were formally documented as pharmacists' interventions (Table 2).

Duplicate therapies were discontinued in 71 (19.6%) of interventions by IV staff compared to 15.6% (n = 7) of interventions by clinical pharmacists. Incomplete prescriptions were addressed in 16 interventions (4.4%) made by IV staff compared to 6 (13.3%) by clinical pharmacists. Drug information inquiries were reported by 4.1% of IV staff interventions compared to 13.3% of clinical pharmacists’ interventions. Interventions related to contraindications or safety were documented in 6 instances (1.7%) by IV staff and in 3 instances (6.7%) by clinical pharmacists. Lastly, interventions related to appropriateness of therapy were conducted by clinical pharmacists in 9 (30%) instances compared to 7 (1.9%) from IV staff.

**Figure 2.**
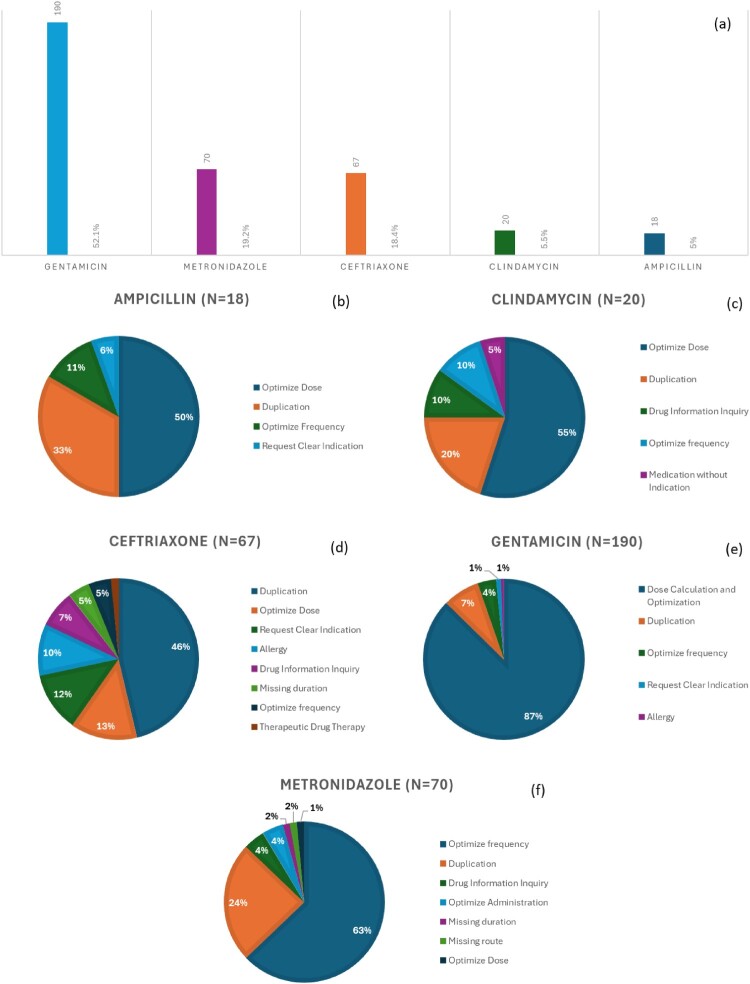
Pharmacists’ interventions on IV antibiotics utilised in labour room. (a) Frequency of reported interventions on used antibiotics; (b) types of interventions on ampicillin prescriptions; (c) types of interventions on clindamycin; (d) types of interventions on ceftriaxone; (e) types of interventions on gentamicin; (f) types of interventions on metronidazole.

Figure 2 illustrates the distribution of intervention types across the IV antibiotics evaluated. Gentamicin had the majority of interventions (n = 190, 52.1%), 87% of which were related to dose calculation and optimisation. Metronidazole was the second most addressed antibiotic (n = 70, 19.2%), and optimising its frequency was the most commonly reported type of intervention (63%). Ceftriaxone was addressed by 67 (18.4%) interventions, mainly for duplication of order (46%), optimising dose (13%), and requesting a clear indication (12%). Clindamycin was addressed in 30 (5.5%) interventions, primarily for optimisation of dose (55%) and duplication of orders (20%). Lastly, 18 (5%) interventions were done on ampicillin, the majority of which were for dose optimisation (50%), followed by order duplication (30%).

## 4. Discussion

### 4.1. Key findings

The study included 464 pregnant patients with an average weight and height of 83.5 (9.7) Kg and 158.9 (4.4) cm, respectively. The majority of the cohort were of Arabic-speaking nationality (70.5%), and 63.1% were primigravida (n = 293). The mode of delivery performed ranged from caesarean section (34.9%) and normal delivery (31.5%) to assisted normal delivery (22.8% vacuum-assisted, 9.7% forceps-assisted). Despite reporting 460 cases of suspected chorioamnionitis, only 376 (81.7%) of the cases met the criteria for diagnosis, with maternal fever, maternal tachycardia, and leukocytosis being the most reported manifestations (83.1%, 79.1%, and 76.1%, respectively).

All patients received gentamicin, 97.8% received ampicillin (mean duration 2.8 ± 0.7 days), 15.5% received clindamycin (0.5 ± 0.8 days), 20.5% received metronidazole (2.5 ± 1.7 days), and 3.4% received ceftriaxone (2.4 ± 1.2 days).

The most common type of intervention performed by IV staff and clinical pharmacists was ‘dosing administration' (68.2% and 31.1%). Notably, the dose of gentamicin was corrected in 306 prescriptions out of the 464 (65.9%), out of which pharmacists actively reported only 175 (57.2%) interventions.

### 4.2 . Interpretation in the context of the wider literature

#### 4.2.1. Appropriateness of chorioamnionitis diagnosis

Evaluation of the diagnostic criteria of presumptive chorioamnionitis yielded a notable discrepancy in reporting, where 16.9% of patients lacked a recording of fever, and 18.3% did not meet the diagnostic criteria. While initiating presumptive treatment for an isolated persistent maternal fever in the presence of other significant risk factors has been suggested, given its association with short- and long-term neonatal complications (Committee Opinion No. 712, 2017; Higgins et al., 2016). Our findings indicate that treatment was initiated in patients lacking reports of fever. This raises concerns about potential overcautious prescribing and unnecessary antibiotic exposure. On another note, retrieving clinical findings from patients’ charts was challenging due to inconsistent documentation practices, potentially leading to misidentification on our part. Improved communication with labour and delivery teams is warranted to better understand the diagnostic reasoning and prescribing behaviour, as well as to enhance the consistency and clarity of clinical symptoms reporting.

#### 4.2.2. Prescription patterns of IV antibiotics for chorioamnionitis

Ampicillin (2gm every 6 h) and gentamicin (1.5 mg/kg/dose every 8 h) were the main regimen initiated in our hospital, in accordance with local and international guidelines (Committee Opinion No. 712, 2017, Lyell et al., 2010; Hamad Medical Corporation, 2025). Clindamycin was the primary agent used for anaerobic coverage before incision for c-section; however, metronidazole was occasionally prescribed post-surgery. It was noted that ceftriaxone was sometimes used during hospital stay for indications other than chorioamnionitis.

Treatment duration after delivery ranged between 2.5 and 3 days, which is the typical turnaround time for culture results at our institution. Only 10 patients completed an IV course of ampicillin/gentamicin therapy (4-7 days). This practice diverges from current evidence, which indicates no benefits of antibiotic continuation following delivery (Committee Opinion No. 712, 2017; Lyell et al., 2010; Shanks et al., 2016). Similarly, local guidelines do not support prolonging antibiotic therapy post-delivery, capping it at 48 h from initiation of therapy, which suggests a misalignment with local and international guidelines.

Our study reports on potentially inappropriate use of antibiotics peripartum, specifically with dosing, duration, and spectrum appropriateness. Similar to our study, inappropriate use of antibiotics in various surgical specialties has been reported (Bedir et al., 2020; Cabral et al., 2023; Ierano et al., 2019; Ou et al., 2014). In an Australian study evaluating surgical antimicrobial prophylaxis (SAP) practices, inappropriate use of prophylactic antibiotics was the major reason for inappropriateness (61.5%) (Ierano et al., 2019). Out of the remaining 1077 post-surgical prescriptions, extended duration was the most common reason for inappropriateness post-procedure (53.4%) (Ierano et al., 2019). Another Chinese prospective study assessed the quality of SAP practices in clean and clean-contaminated procedures and used orthopaedic surgeries as the reference group (Ou et al., 2014). It concluded that gynecological procedures demonstrated significantly high rates of inappropriateness compared to orthopaedic procedures (odds ratio, 1.60; 95% CI, 1.37-1.88; *P* < .001) (Ou et al., 2014). The abundance of available evidence supporting similar patterns to those observed in our study warrants an urgent need for practical interventions to increase adherence and appropriateness of antibiotic usage.

Our study did not assess clinical outcomes to elucidate the maternal and neonatal benefits or risks of prolonged antimicrobial therapy; however, there is a strong body of evidence associating unnecessary antibiotic continuation with increased antimicrobial resistance (Ho et al., 2025; Vitiello et al., 2024). The discrepancy in practice necessitates the need for direct discussion with the multidisciplinary team to better understand prescribing behaviours and promote alignment with guideline-concordant practice.

#### 4.2.3. Role of pharmacists in the management of IV antibiotics used in treatment

To the best of our knowledge, this is the first study to highlight the significant role of IV staff pharmacists in labour and delivery departments, demonstrating their proactive involvement in rectifying IV antibiotic orders, particularly with regard to dosing and administration of antibiotics. While literature has established the clinical pharmacists’ roles in obstetrics and gynaecology departments locally (AlSaad et al., 2021) and internationally (Naseralallah, et al., 2024; Marino-Martinez et al., 2016), there is a paucity of literature investigating the role of staff pharmacists, especially with regard to preparation and administration of IV therapies.

One study has reported a prospective aminoglycoside initiation audit performed by staff pharmacists, which concluded a successful implementation and attainment of interpretable therapeutic, non-toxic serum levels in 71.4% of reviewed cases (Possidente & Lynch, 1988). Similarly, a study by Cheon et al. investigated the impact of incorporating an IV drug prescription review into hospital pharmacist s’ work scope, concluding that the new task could be performed in a timely manner to enable identification of incompatibility risk of IV pairs and prevent associated risk (Cheon et al., 2023).

Our study illustrates the active involvement of IV staff pharmacists in optimising patient care through timely interventions addressing dose adjustments, administration techniques, clarification of contraindication status, and confirmation of appropriate therapy. Specifically, this cohort illustrates a repetitive trend of intervening to tailor gentamicin dose to patient weight. Given the need to contact prescribers to amend orders, delays in medication provision are anticipated. A similar trend was reported in a study assessing the effect of pharmacist-to-dose electronic requests on the promptness of antibiotic therapy, where the reduction in medication errors was accompanied by delays in verifying and releasing the first doses of vancomycin and gentamicin (Vincent et al., 2009). These findings illustrate the urgent need for targeted education and dissemination of prescribing guidance to prevent prescribing errors resulting in medications’ delays.

Pharmacists’ role in prospective auditing and intervening for antibiotic dose adjustments is well established in literature (Gatechan et al., 2025; Hirano et al., 2016; Powell & Lantos, 1981; Vincent et al., 2009), forming a core component of antimicrobial stewardship (AMS) programmes. Several studies have demonstrated the effectiveness of pharmacist-led AMS programmes (Cantudo-Cuenca et al., 2022; Naseralallah et al., 2024; Sawada et al., 2023). A meta-analysis assessing the effectiveness of pharmacist-led AMS programmes in perioperative settings (including obstetrics and gynaecology) concluded a significant improvement in antibiotic selection (OR 4.29; 95% CI 2.52–7.30), administration time (OR 4.93; 95% CI 2.05–11.84), duration (OR 5.27; 95% CI 1.58–17.55), and reduction of surgical site infections (OR 0.51; 95% CI 0.34–0.77) (Naseralallah et al., 2024). Additionally, AMS programmes led by pharmacists in hospitals with no infectious disease specialists or pharmacists have shown successful implementation, resulting in a significant reduction in antibiotics’ consumption and expenditure (Cantudo-Cuenca et al., 2022; Sawada et al., 2023), length of stay, duration of therapy, empirical use of certain antibiotics, and time to de-escalation (Sawada et al., 2023).

While our study did not report on clinical outcomes, it provides valuable insights into existing institutional practices and highlights the critical, proactive role that staff pharmacists play in mitigating antimicrobial therapy errors.

### 4.3. Future directions

**Figure 3.**
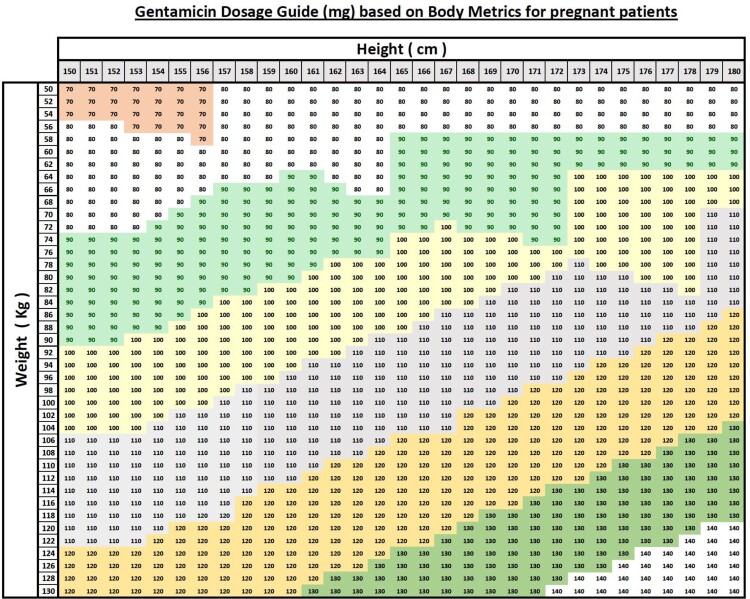
IV pharmacy team initiative to tackle the incorrect dosing of gentamicin.

The results of this study highlight the need for revision of current practices in the labour and delivery department and reassembling with the medical team to ensure alignment with evidence-based guidelines. In light of the delays observed in antibiotic initiation due to gentamicin dose calculation, we developed a proposed checklist to facilitate timely and appropriate recognition of suspected chorioamnionitis (Supplemental Material), as well as a dosing algorithm to streamline accurate gentamicin prescribing (Figure 3). Although these tools were not tested in this study, they provide a structured framework for clinical decision-making and merit assessment in future quality improvement initiatives following their implementation.

Additionally, future studies should focus on assessing the impact of pharmacist -led interventions on IV antibiotics use in this cohort, including maternal and neonatal outcomes and potential reductions in prescribing errors and therapy delays.

### 4.4. Strengths and limitations

This study was conducted in the largest tertiary hospital providing antenatal and postnatal care in Qatar, providing substantial insight into current national practices. It adds to the literature by capturing real-world data on the types of IV antibiotics used for chorioamnionitis and highlighting the pivotal role of IV staff pharmacists in optimising antimicrobial therapy. Additionally, this is the first study in the region to shed light on the practice of staff hospital pharmacists and their role with IV admixtures, specifically antibiotics.

However, several limitations should be acknowledged. The retrospective design inherently depends on the accuracy and completeness of routine clinical documentation, which may introduce information bias. The single-centre nature of the study may limit generalizability and may not fully capture variability in practice across other hospitals in Qatar. Furthermore, this study was descriptive in nature and did not follow up patients to evaluate the impact of practice on maternal and fetal outcomes. To address these limitations and establish a more robust overview of current practices, future studies should incorporate data from all hospitals handling labour and delivery and consider prospective designs that evaluate the effect of pharmacist-led interventions on clinical outcomes and antimicrobial stewardship metrics.

## 5. Conclusion

This study highlights the role of staff IV pharmacists in managing IV antibiotics utilised in the setting of chorioamnionitis. Observed deviations from recommended regional and global guideline-concordant antibiotic use reveal opportunities for system-level optimisation through synchronised multidisciplinary action. The findings underscore pharmacists’ crucial preventive and corrective contributions in intercepting medication-related inaccuracies and advancing sustainable error-reduction strategies to elevate obstetric antimicrobial stewardship and patient safety.

## Supplementary Material

Supplemental Material
