# Supplementary material for: Use of intravenous antibiotics for the management of suspected chorioamnionitis: insights from women's wellness and research center
Source: J Pharm Policy Pract. 2026 May 7;19(1):2664880. doi: 10.1080/20523211.2026.2664880 (PMC13159593; doi:10.1080/20523211.2026.2664880)
Supplement: Supplemental Material [file JPPP_A_2664880_SM8755.docx]

**Chorioamnionitis Clinical Diagnostic Criteria**

**Fever (**≥**37.8 °C)**


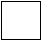
 No fever recorded


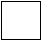
 One spike of fever ≥37.8 °C


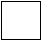
 Recurrent fever ≥2 spikes ≥37.8 °C


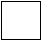
 Persistent fever >38 °C over ≥2 readings

**Maternal Tachycardia (>100 bpm)**


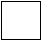
 HR normal (<100 bpm)


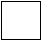
 HR >100 bpm


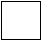
 Sustained tachycardia >100 bpm for >30 minutes


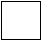
 Associated with fever [ ] Yes [ ] No

**Leukocytosis (WBC >15,000/mm³)**


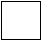
 WBC normal <15,000


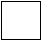
 WBC elevated >15,000

**Fetal Tachycardia (>160 bpm)**


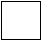
 Normal FHR (<160 bpm baseline)


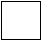
 FHR >160 bpm


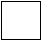
 Sustained >10 minutes (Yes / No)


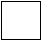
 Associated with maternal fever (Yes / No)

**Foul-Smelling Amniotic Fluid**


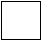
 Not applicable (membranes intact)


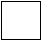
 Amniotic fluid observed


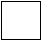
 Clear


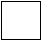
 Foul odor present (describe: ____________)

**Uterine Tenderness**


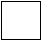
 No tenderness on palpation


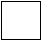
 Mild tenderness


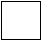
 Moderate tenderness


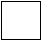
 Severe tenderness

- Notes: ______________________________________

**Diagnosis Summary**


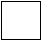
 Criteria met for suspected clinical chorioamnionitis (≥1 fever + ≥1 additional findings)


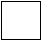
 Criteria not met
